# Sphingosine-1 phosphate receptor (S1p1), a critical receptor controlling human lymphocyte trafficking, is expressed in hen and human ovaries and ovarian tumors

**DOI:** 10.1186/1757-2215-4-4

**Published:** 2011-02-28

**Authors:** Michael J Bradaric, Animesh Barua, Krishna Penumatsa, Yu Yi, Seby L Edassery, Sameer Sharma, Jacques S Abramowicz, Janice M Bahr, Judith L Luborsky

**Affiliations:** 1 Department of Pharmacology, Rush University Medical Center, Chicago, IL USA; 2 Department of Obstetrics & Gynecology, Rush University Medical Center, Chicago, IL USA; 3 Department of Pathology, Rush University Medical Center, Chicago, IL USA; 4 Department of Obstetrics & Gynecology, Section of Gynecologic Oncology, John Stroger Hospital, Chicago IL USA; 5 Department of Animal Sciences, University of Illinois Urbana - Champaign, IL USA

## Abstract

**Background:**

Sphingosine-1 receptor 1 (S1P1) plays a major role in regulating lymphocyte egress from peripheral lymph tissue. Lymphocyte trafficking is potentially a critical response to tumors and to tumor vaccines. Also, the receptor has been shown to influence metastasis. However, there is little information on its expression in the aged ovary or ovarian tumors. As a basis for further studies in the laying hen model of spontaneous ovarian cancer, the objective of this study was to determine if S1P1 is expressed in hens, and if the morphological distribution of S1P1 is similar in hen and human ovary and ovarian tumors.

**Methods:**

S1P1 mRNA was ascertained in hen tissue by RT-PCR using hen specific primers. S1P1 protein expression and localization was evaluated in hen and human tissue with a human S1P1 antibody by Western blot and immunohistochemistry.

**Results:**

S1P1 mRNA was expressed in all hen tissues examined. Protein was detected in human and hen ovary and ovarian tumors at 47, 72 and 108 kDa in Western blots. S1P1 was similarly expressed on endothelial cells, lymphocytes and surface epithelial cells in normal ovaries and tumor-containing ovaries of the hen. In addition, S1P1 distribution was heterogeneous in both hen and human ovarian tumors by immunohistochemistry.

**Conclusion:**

The results show that S1P1 is expressed in the hen and human ovary as well as in ovarian tumors. These findings support the use of the hen in further studies of the role of S1P1 in metastasis and immune cell trafficking in ovarian tumor development.

## Background

Sphingolipids acting through sphingosine-1-phosphate receptors are involved in embryogenesis, angiogenesis, vascular homeostasis and immune cell trafficking [1,2]. There are five isoforms of sphingosine receptors (S1P1 - S1P5) [3]. Sphingosine receptors are members within a larger family of G-Protein Coupled Receptors (GPCR) that are expressed on leukocytes and on vascular endothelial cells. The ligand, sphingosine-1 phosphate (S1P), binds to several of the sphingosine 1-phosphate receptors with higher affinity to the S1P1 and S1P3 isoforms [4]. The S1P1 regulates lymphocyte egress from lymphoid organs [5,6] and is necessary for lymphocyte recirculation from thymus and peripheral lymphoid organs. In addition to a critical role in regulating immune cell trafficking, activation of S1P1 can promote or inhibit apoptosis of immune cells depending on the balance of cytokines [7]. Knockout of S1P1 (LP(B1)/EDG-1) in mice is embryologically lethal [8]. S1P1 also has a role in inflammatory diseases such as graft versus host disease and multiple sclerosis [9]. The drug FTY720 binds to S1P1 as a high affinity agonist and causes down-regulation and internalization of S1P1. This drug has been used as a novel immunosuppressive agent to inhibit S1P1-mediated immune cell migration from lymph to sites of inflammation and is of particular interest in transplant and in treatment of autoimmune diseases such as multiple sclerosis [9] and more recently, cancer.

The endogenous ligand (S1P) was recently shown to play an important role in ovarian cancer invasiveness and ovarian tumor cell migration [10,11]. It also appears to protect ovaries from the effects of chemotherapy [12] and radiation [13] and, therefore, is potentially a therapeutic target to preserve fertility in patients undergoing therapy for cancer. While there are several studies of S1P involvement in ovarian cancer models and ovarian tumor-derived cell lines there is no information on the expression of its receptor, S1P1, in normal human (aged) ovary or in naturally occurring ovarian tumors in humans or animal models.

We [14-18] and others [19-21] reported that the laying hen, which spontaneously develops ovarian tumors [22] is useful for studies of ovarian cancer. The normal hen ovary has been used extensively to understand ovarian physiology [23,24] because it shares many features of normal human ovary including similar cyclic hormone regulation of follicle development and ovulation [25]. Like human ovaries, hen ovaries express receptors for follicle stimulating hormone (FSH) and luteinizing hormone (LH) and produce inhibins, estrogen, and progesterone in response to FSH and LH [24]. One difference between human and hen ovarian function is the lack of post-ovulatory development of a progesterone-secreting corpus luteum and the events that lead to implantation because eggs are laid externally.

Likewise, naturally occurring hen ovarian tumors are similar to human tumors [17,22]. Commonly, hen ovarian tumors exhibit epithelial cell histology including serous, endometrioid, clear cell and mucinous histology [17] and less frequently tumors of germ cell origin [22] which is typical of the histology seen in humans [26]. The incidence of both hen and human ovarian tumors increases with age [22,27]. In hens, which are pure bred (rather than inbred), the incidence of ovarian tumors is also strain and flock dependent [20] which suggests a genetic component associated with ovarian cancer, as in humans [28]. As well, many of the same proteins are expressed in human and hen tumors such as CA125 [29], E-cadherin [30], COX [19], p53 [28], SBP-1 [31], mesothelin [32] and several others [21]. Interestingly, progesterone reduced the incidence of ovarian carcinoma in hens which parallels the reduced risk of ovarian cancer associated with oral contraceptive use in women [33]. Recently, we developed the use of ultrasound to assess ovarian morphology and tumor-associated angiogenesis [18] in order to facilitate the selection of hens for studies of ovarian cancer and to be able to monitor hens longitudinally.

A further advantage of the hen as a model for studies of immune mechanisms in ovarian cancer is the well established knowledge of the hen immune system. In fact, the two different types of immune cells (T and B cells) were first described based on the differences in lymphocytes in the thymus and bursa of Fabricius [34,35]. Also, the first successful anti-tumor vaccine was developed for chickens to prevent Marek's disease, a virally-induced lymphoid neoplasm [36]. Moreover, humans [37,38] and hens [16] develop spontaneous ovarian autoimmunity and circulating anti-ovarian antibodies associated with prematurely reduced ovarian function.

Our future objective is to examine the role of immunity in ovarian tumor development and progression through modification of lymphocyte trafficking. Although the expression and role of S1P1 has been demonstrated in humans, there is little information on its expression in the human or hen ovary. Therefore, the specific objective of this study was to determine if S1P1, a major receptor that regulates lymphocyte trafficking in humans, is expressed in hens, and if the morphological distribution of S1P1 is similar in hen and human ovary and ovarian tumors.

## Methods

### Animals

White leghorn hens (2-3 years old, strain W/96) were housed at the University of Illinois at Urbana-Champaign (UIUC) at the Poultry Research Farm affiliated with the Department of Animal Science. Food and water were given *ad libitum *and hens were maintained on a 17:7 hour light: dark schedule. Hens this age were used in our study because the proportion of hens with ovarian tumors is about 10-15%, based on our experience. Animals were selected for study based on normal or abnormal ovarian ultrasound as described previously [15,17,18]. Hens were sacrificed at UIUC by cervical dislocation and organs removed. Hen ovaries (n = 30) were histologically staged and typed by a pathologist using criteria similar to human tumor type and staging as described previously [17]. All procedures were approved by the University of Illinois Institutional Animal Care and Use Committee (IACUC).

### Human Ovarian Tissues

Normal ovaries and ovarian tumors were obtained from the gynecologic oncology clinics at Rush University Medical Center and John Stroger Hospital (Chicago, IL) according to Institutional Review Board (IRB) approved protocols. The criterion for inclusion in the study was women ≥ 45 years old. The criteria for exclusion were a previous history of any cancer and prior chemotherapy or radiation treatment. Normal ovaries were obtained at hysterectomy (n = 5; mean age 54 ± 8 years). Ovarian tumors were obtained from patients with malignant tumors (n = 5; mean age 64 ± 15 years). The tumor histology and tumor grade were determined by a pathologist using standard FIGO criteria [17]. Of the five ovarian tumors shown in this report, three were serous and two were endometrioid.

### Tissue preparation

Hen ovary (n = 30), spleen (n = 5), and caecal tonsils (peripheral lymphoid organ, n = 4) and brain (n = 2) were cut into three equal portions. There were 11 normal ovaries and 19 ovarian tumors used for these experiments. Tissues were prepared for histological and biochemical analysis. All ovarian tissue was examined to verify normal or tumor histology (n = 30). For immunohistochemical analysis, 23 tissues were used and for Western blot and PCR, 20 and 30 tissues were used, respectively. Human (normal ovary, n = 5) and ovarian tumors (n = 5) were similarly prepared. One portion was fixed in 10% PBS-buffered formalin and embedded in paraffin for histology and immunohistochemistry [17]. Sections of formalin-fixed, paraffin-embedded tissue stained with Hematoxylin and Eosin (H/E) were examined by a pathologist to determine the histological type and stage. A second portion was frozen (-80°C) for cryostat sections for immunohistochemistry. The final portion was washed with cold 1.5 mM Tris HCl, homogenized (100 mg wet weight tissue/100mL of 40 mM Tris HCl, 5 mM MgSO_4 _buffer), centrifuged (1,000 × g, 10 minutes, 4°C) and the supernatant stored at -80°C for Western blot analysis [16,31]. In addition, to enrich for S1P1 receptors, the supernatant was centrifuged again (18,000 × g, 40 minutes, 4°C) and the pellet was suspended in sample buffer (Bio-Rad Laboratories, Hercules, CA) for one-dimensional gel electrophoresis (1D-PAGE). Rat brain was used for control and was a gift from Dr. Amanda Mickiewicz (Rush University, Chicago).

### Reverse transcription-polymerase chain reaction (RT-PCR)

To assess S1P1 mRNA expression, RT-PCR was performed as reported previously [38]. Briefly, total RNA from 30 ovaries (11 normal and 19 tumor) and 14 organs was extracted using Trizol reagent (Invitrogen, Carlsbad, CA). The RNA content was measured at an optical density (OD) of 260 nm and the purity evaluated using an OD 260/280 nm absorbance ratio ≥ 1.7. RNA was treated with DNASe (Invitrogen, Carlsbad, CA) to remove trace amounts of genomic DNA before the first strand synthesis. First strand synthesis was performed using 500 ng of RNA according to the manufacturer's protocol (37°C, 1 hour; High Capacity cDNA RT Kit, (Applied Biosystems, Carlsbad, CA). The PCR amplifications were carried out in a 25 μl reaction volume containing 25 ng of cDNA using Platinum Taq DNA Polymerase (Invitrogen, Carlsbad, CA) according to the manufacturer's recommendation. The PCR cycle consisted of a primary denaturation at 94°C (3 minutes) followed by 35 cycles of denaturation at 94°C (30 seconds) and 54°C (30 seconds) to anneal and 72°C (1 minute) for extension followed by a final extension at 72°C (10 minutes) in a programmable Peltier Thermo Cycler (PTC-200, MJ Research Inc., Ramsey, MN). Hen-specific S1P1 primers were designed using Oligoperfect Designer software (Invitrogen, Carlsbad, CA) using the S1P1 sequence from the NCBI [GeneBank: XM_422305.2]. The forward primer was CCCCAGGAGCATTAAAACTG and the reverse primer was CTGCTGACCACCCTCACTG located between exons 1 and 2. β-actin was used as the endogenous control with a forward primer of TGCGTGACATCAAGGAGAAG and a reverse primer of ATGCCAGGGTACATTGTGGT. The expected base pair size for the S1P1 amplicon was 226 bp and for β-actin was 300 bp. PCR amplicons were visualized in a 2% agarose gel (Pierce/Thermo Fisher, Rockford, IL USA) in T.A.E. buffer (4.84g Tris Base, 1.14mL acetic acid, 2.0 mL 0.5M EDTA/L of buffer) and stained with ethidium bromide. The image was captured using a ChemiDoc XRS system (Bio-Rad, Hercules, CA). Amplicon from a positive sample (endometrioid carcinoma of the ovary) was used for sequence analysis after purification using the Quia-Quick PCR Purification System (Qiagen, Valencia, CA USA) according to manufacturer's instructions. The purified DNA was sequenced at the DNA sequencing facility at the University of Illinois at Chicago using an ABI 3100 Genetic analyzer (Applied Biosystems, Foster City, CA).

### One-dimensional (1D) Western Blot

Some ovarian tissue samples (n = 20; 9 normal, 11 tumor) were homogenized according to a previous protocol [39] and stored at -80°C. Proteins (10 μg/lane) were separated by 1D gel electrophoresis using 10% gradient Tris-HCl gels (Bio-Rad, Hercules, CA) using standard procedures [31]. MagicMark XP Western blot standards (Invitrogen, Carlsbad, CA) were used to estimate molecular weight. Rat brain (n = 3) was used as a positive control (recommended by Cayman Chemical website). Proteins were transferred (18 Volts, 30 minutes) to a nitrocellulose membrane (0.45 μm; Bio-Rad, Hercules, CA). Blots were blocked in 10 × Blocking Buffer (diluted to 1×; Sigma St. Louis, MO) containing 0.05% Tween-20 (4°C; 16 hours; Sigma, St. Louis, MO), rinsed in Wash buffer (0.15 M NaCl in 10 mM Tris containing 0.05% Tween-20, pH7.5) and incubated in rabbit anti-S1P1 polyclonal antibody (1:200; Cayman Chemical, Anne Arbor MI) diluted in blocking buffer containing 0.05% Tween-20. The nitrocellulose membrane was washed three times in cold Wash buffer followed by goat anti-rabbit immunoglobulin-HRP (Horseradish - Peroxidase; Pierce/Thermo Fisher, Rockford, IL). As a control for antibody specificity the anti-S1P1 antibody was pre-absorbed with blocking peptide (Cayman, Ann Arbor, MI) (1:1, v/v; 45 minutes, 22°C). The absorbed, control anti-S1P1 was diluted to the same concentration as the untreated S1P1 antibody (1:200) in blocking buffer (Sigma, St. Louis MO) supplemented with 0.05% Tween-20 and used as primary antibody. The reaction was developed in Super Signal West Dura substrate (Pierce/Thermo Fisher, Rockford, IL) and digital images acquired using a ChemiDoc XRS system (Bio-Rad, Hercules, CA). Digital images were analyzed by Quantity One software (Bio-Rad, Hercules, CA).

Because there are currently no commercially available antibodies against avian S1P1, we used a commercially available polyclonal antibody against human S1P1 for Western blotting and immunohistochemical experiments. There are two serine (S) to threonine (T) substitutions in the chicken S1P1R, within amino acids 241-253 of the epitope, and a high degree of homology (> 85% based on sequence comparisons) between the two proteins.

### Immunohistochemistry

For cryostat sections, tissue was washed in cold phosphate buffered saline (PBS, pH 7.0) and placed in 30% sucrose overnight at 4°C. Tissues were washed once more in PBS the following morning, embedded in OCT Compound (Tissue Tek, Sakura, Japan) and flash frozen in dry-ice cooled methanol and stored at -80°C until use.

Ovarian sections were incubated with rabbit anti-S1P1 (Cayman, Ann Arbor, MI) diluted 1:200 in PBS containing 1% BSA (bovine serum albumin; Fisher, Waltham, MA). The primary antibody was omitted as a control for non-specific antibody binding. Other primary antibodies for immune cell markers include Bu1a (chB6; Abcam, Cambridge, MA) and T cell antibodies (CD3, CD4, and CD8; Southern Biotech, Birmingham, AL). As a control for antibody specificity the anti-S1P1 was pre-absorbed with blocking peptide (Cayman, Ann Arbor, MI) (1:1, v/v; 45 minutes, 22°C). The absorbed, control anti-S1P1 was diluted to the same concentration as the untreated S1P1 antibody (1:200) in normal horse serum and used as primary antibody. Sections were washed and incubated with goat anti-rabbit immunoglobulin-HRP (Pierce/Thermo Fisher, Rockford, IL) (1:10,000 in Sigma Blocking Buffer containing 0.05% Tween-20;1 hour; 22°C; Sigma, St. Louis, MO). Color was developed with 3, 3-diaminobenzidine (DAB) substrate (Vector Labs; Burlingame, CA). Slides were washed in running water (15 minutes) and counterstained with hematoxylin followed by dehydration with graded alcohol series (70 -100%) and xylene. Sections were examined with an Olympus light microscope (BX41, Tokyo, Japan) and an Olympus U-CMAD3 camera with Micro Suite #5 software.

## Results

### S1P1 mRNA is expressed in hen tissues

**Figure 1:**
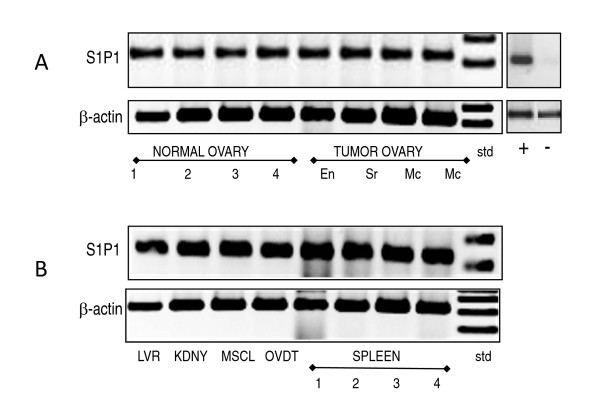
**S1P1 mRNA expression in hen tissues**. (**A**) S1P1 mRNA (226 bp) is expressed in both normal and tumor ovaries. Examples of mRNA in tumors with endometrioid (En), serous (Sr), and mucinous (Mc) histology are shown. (**B**) Examples of other hen tissues that express S1P1 mRNA (226 bp) include liver (LVR), kidney (KDNY), skeletal muscle (MSCL), oviduct (OVDT) and spleen. Normal ovary and spleen are from the same hens (1-4). β-actin (300 bp) was used as a loading control. Controls for S1P1 primer include the positive control (+) lane which was the sample from an earlier experiment used to verify the RNA sequence. The negative control lane omitted the cDNA.

The mRNA for S1P1 was detected at the predicted amplicon size of 226 bp in hen tissue (Figure 1). Four normal ovaries (no evidence of cancer) and four tumor ovaries with endometrioid, serous and mucinous histology had S1P1 mRNA (Figure 1A). Other tissues, including muscle, oviduct, liver and kidney also contained S1P1 mRNA (Figure 1B). The expression of S1P1 mRNA was confirmed by sequence analysis at University of Illinois at Chicago DNA Services Facility (DNAS) and was the positive control shown in Figure 1. The negative control lane omitted the use of cDNA. Human tissue was not evaluated for S1P1 mRNA expression because it was demonstrated previously [40].

### S1P1 protein is expressed in hen tissues

**Figure 2:**
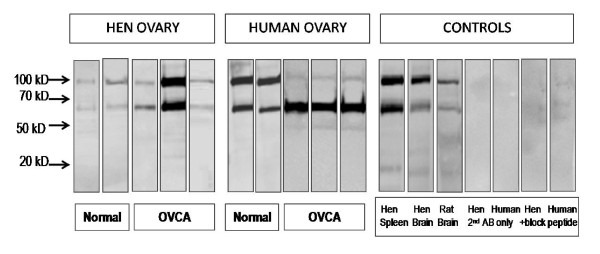
**S1P1 protein expression in hen and human tissue**. S1P1 immunoreactions are similar in hen and human ovaries and ovarian tumors. Three bands at 47, 72, 108 kDa were observed. The band at 47 kDa was faint, while bands at 72 and 108 kDa were consistently present in all tissues but vary in intensity. The 47 kD band was not significantly enhanced using a membrane enriched (18,000 × g pellet) fraction. The pattern of immunoreactive bands was identical in the positive control recommended by the manufacturer (rat brain) and in hen brain and spleen. The bands were absent in control incubations in which the primary antibody was pre-adsorbed with a blocking peptide or in which the primary antibody was omitted.

S1P1 protein was expressed in human and hen ovaries and ovarian tumors with bands at 47, 72 and 108 kDa detected by Western blot (Figure 2). There were variations in the intensity of bands at each molecular size from different preparations in both hen and human tissues. A membrane-enriched fractionation (18,000 × g) did not result in a consistently enhanced 47 kDa band in either the hen tissues or control rat brain. Hen brain showed the same bands as the positive control. Spleen was expected to express S1P1 because it is a major lymphocyte processing organ and the Western blot reactions were the same as the rat and hen brain. The band intensity was reduced using anti-S1P1 antibody pre-absorbed with blocking peptide and was absent when the primary antibody was omitted.

### S1P1 localization in hen ovaries and ovarian tumors by immunohistochemistry

**Figure 3:**
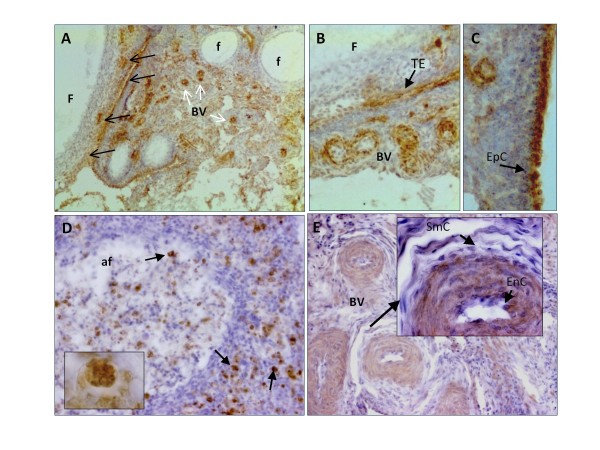
**Localization of S1P1 receptor protein expression in normal hen ovary**. (**A**) S1P1+ cells (black arrows) in theca of a mature follicle (F) and within small blood vessels (BV; white arrows) in ovarian stroma (100×). Primordial follicles (f) have comparatively little S1P1+ expression. (**B**) Endothelial cells of blood vessels (BV) in the theca externa (TE) of a follicle (F) and ovarian stroma are S1P1+ (200×). (**C**) Surface epithelial cells (EpC) showing intense S1P1+ expression (400×). (**D**) An atretic follicle (af) with characteristic infiltrating S1P1+ immune cells (100×). Inset: High magnification of (D) showing an S1P1+ immune cell (1000×). (**E**) Well developed blood vessels (BV) in the medullary region of the ovary also contain S1P1+ endothelial cells (400×) but staining is lighter and more diffuse than in stromal blood vessels seen in (A) and (B). Inset: high magnification (800×) shows detail of smooth muscle cells (SmC) and endothelial cells (EnC). (**A-C**) are frozen tissues; (**D**) and (**E**) are paraffin-embedded.

S1P1 was expressed in normal hen ovaries in blood vessels in the stromal (Figure 3A and 3B) and medullary regions (Figure 3E) of the ovary. S1P1 was also found in mature follicles, but not in early stage follicles (Figure 3A). Within mature follicles, S1P1 was expressed exclusively in the theca externa (Figure 3A). Surface epithelial cells of the ovary also showed intense S1P1 expression (Figure 3C). Atretic follicles (Figure 3D) had S1P1+ immune cells (insert) but S1P1 staining was absent in follicle remnants. The endothelial cells but not the smooth muscle cells of blood vessels were S1P1+ (Figure 3E, insert).

**Figure 4:**
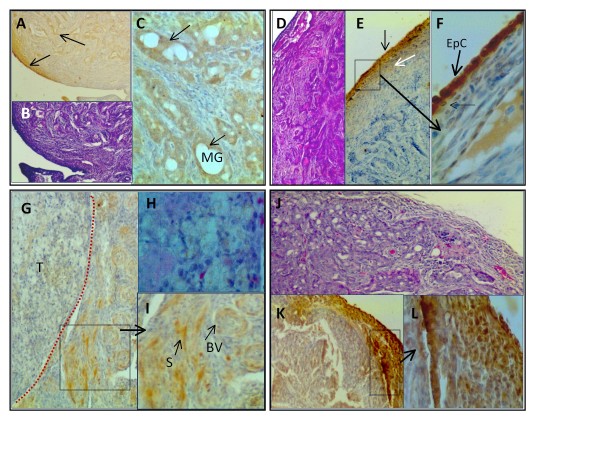
**Localization of S1P1 receptor protein expression in hen ovarian tumors**. (**A**) An example of a mucinous ovarian tumor with S1P1+ staining associated with surface epithelium (black arrow) and mucin-secreting structures (black arrow) (100×). (**B**) H& E stained section (100×) serial to that in (A) shows mucinous histology. (**C**) Higher magnification of (A) showing S1P1+ mucin-secreting glandular (MG) structures (black arrows) (400×). (**D**) H&E stained section of a serous ovarian tumor (100×). (**E**) Serial section showing minimal stromal cell stain for S1P1+ but intense surface epithelial cell staining (black arrow) and lighter more diffuse S1P1+ sub-epithelial cells (white arrow) (100×). (**F**) High magnification (of box) showing S1P1+ surface epithelial cells (EpC) (600×). (**G**) Clear-cell ovarian tumor (T; left of dotted red line) with negligible S1P1+ in tumor and S1P1+ cells in adjacent uninvolved stroma (100×). (**H**) H&E-stained serial section from the same tumor region in (G) shows cellular detail of clear cell carcinoma (400×). (**I**) Higher magnification of box in(G) showing stromal blood vessels (BV) with S1P1+ endothelial cells and stromal cells (S) (200×). (**J**) H&E stained section of late stage endometrioid tumor (100×). (**K**) S1P1+ is highly expressed in cells in the tumor periphery and to a lesser extent in tumor stroma (100×). (**L**) High magnification of box in (K) showing cytoplasmic staining of endometrioid tumor cells (400x). All images are from paraffin-embedded tissue, except clear cell carcinoma (G-I) which is a frozen section.

Hen ovarian tumors had varied S1P1 staining (Figure 4). A mucinous ovarian tumor had S1P1 staining associated with mucin-secreting glandular structures (Figure 4A and 4C). An example of a serous ovarian tumor shows light stromal cell cytoplasmic S1P1 staining but intense staining of the surface epithelium (Figure 4E and 4F). Endometrioid (Figure 4K and 4L) ovarian tumors had similar S1P1+ staining within the tumor; the most intense staining being associated with surface epithelial cells and the area immediately adjacent to it (Figure 4L). Most of the S1P1+ cells associated with clear cell carcinomas were outside the tumor (Figure 4G), while blood vessels in the uninvolved stroma adjacent to the tumor were S1P1+ (Figure 4I).

### S1P1 localization in human ovary and ovarian tumors by immunohistochemistry

**Figure 5:**
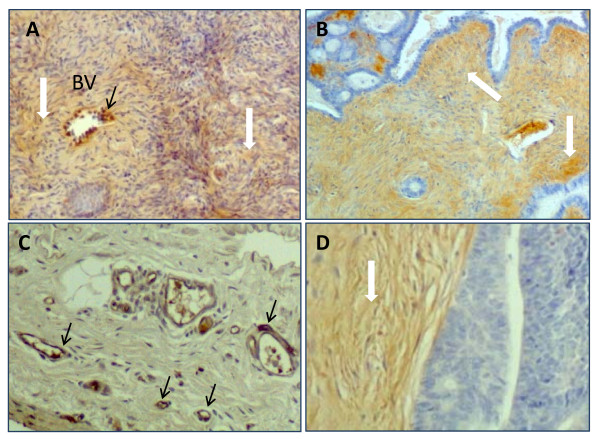
**S1P1 receptor expression in human ovarian carcinomas**. **(A)**	Normal ovary showing diffusely stained S1P+ stromal cells (white arrows) and a blood vessel (BV) with intensely stained S1P1+ endothelial cells (black arrow) (200×). **(B) **Serous ovarian tumor with S1P1+ stroma (white arrows) and unstained surface epithelium (200×). **(C) **blood vessels in normal ovary (BV, arrows) are S1P1 + (600×). **(D) **S1P1+ endometrioid ovarian tumor with patches of S1P1+staining within the surrounding stroma (white arrows) adjacent to unstained endometrioid tumor (400×). (A-D) are paraffin-embedded sections.

The staining patterns of S1P1 in human ovarian cancers were heterogeneous, similar to the hen ovarian tumors. Normal ovaries had endothelial cell S1P1 staining around blood vessels as well as light staining of the ovarian stroma (Figure 5A and 5C). Serous ovarian tumors had S1P1 staining in the stroma but not the epithelium (Figure 5B). Endometrioid tumor structures were not stained, but surrounding stroma was S1P1 immuno-stained (Figure 5D).

### S1P1 expression associated with immune cells in ovaries of hens

**Figure 6:**
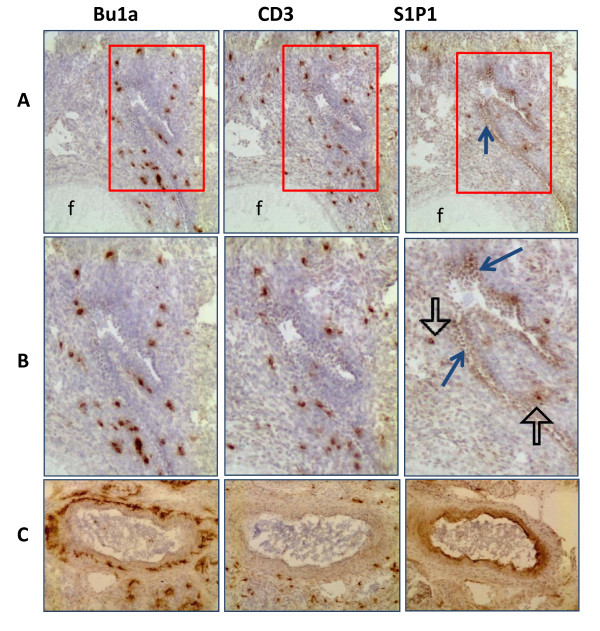
**In normal hen ovary S1P1 expression was observed in areas of immune cell infiltration**. (**Row A) **Immune cells (Bu1a+ and CD3+) are adjacent to the follicle (f) in the ovarian stroma near a transverse blood vessel (arrow in column S1P1). Cells lining the vessel near the follicle (f) are S1P1+ (row A, S1P1) (original magnification 100×). (**Row B) **High magnification (see red boxes in row A) showing B and T cells clustered within the stroma near S1P1 stained vascular endothelium (arrow). Some immune cells are also S1P1+ (open arrow) (original magnification 400×). (**Row C) **Cross-section of a large blood vessel shows Bu1a and CD3 positive cells are clustered near the blood vessel. The apical surfaces of endothelial cell express S1P1 (original magnification 100×).

**Figure 7:**
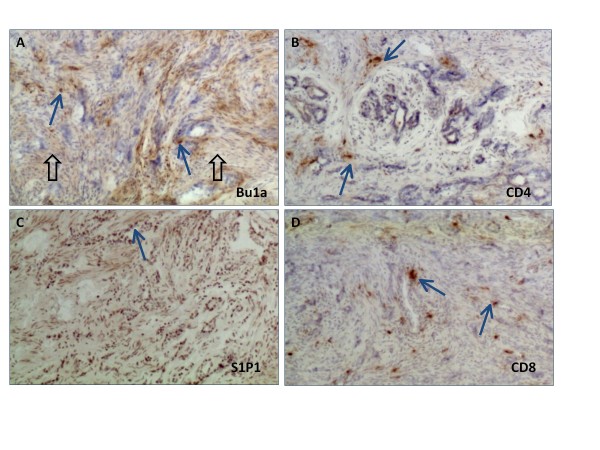
**In ovarian tumors S1P1 expression was observed near T and B cells**. Alternate serial sections of a serous ovarian tumor of the hen showing (**A) **Bu1a+ cells in the stroma (arrows) and diffuse tissue stain (open arrow), (**B) **CD4 T cells around tumor glands (arrows), (**C) **S1P1 expression on the epithelium of tumor glands (arrow) and (**D) **CD8 T cells (arrows) in the stroma (original magnification 100×).

Serial frozen sections of ovarian tissue were stained with hen specific antibodies against Bu1a (antigen specific for avian B cells) and CD3 to determine if S1P1 expression was associated with immune cells (Figures 6 and 7). In normal ovaries (Figure 6), S1P1 was expressed on cells both with and without B or T cell markers in the ovarian stroma and was primarily expressed on blood vessels. The B and T cells were found in close proximity to S1P1 stained blood vessels. In tumors (Figure 7) staining patterns were less organized. S1P1 staining occurred in serous tumor cells. While CD4 T cells were more often found scattered around the tumor glands, CD8 T cells and Bu1a+ staining was localized throughout the tissue and in tumor glands.

## Discussion

This is the first study reporting the expression of S1P1 in ovarian tissues in the adult laying hen. Although chicken specific primers were used to detect S1P1 mRNA and an anti-human S1P1 antibody was used to detect S1P1 protein, the expression of S1P1 mRNA and protein were correlated. Similarly, S1P1 was detected by immunohistochemistry in tissue positive for S1P1 mRNA and protein. This is consistent with the high degree of amino acid similarity (> 85%) between avian [GenBank ACC#: XP_001231780.1] and human [GenBank ACC#: NP_001391.2] S1P1 protein. Furthermore the location of S1P1 positive cells was similar in hen and human. In normal ovaries and ovarian tumors, S1P1 was expressed in endothelial cells of blood vessels and immune cells. In follicle cells of normal hen ovary, theca externa cells but not ovarian stroma nor other follicular structures were stained. Follicles in normal human ovary were not observed in this study because tissue from post-menopausal women was used and thus it was not possible to compare them with the hen follicles. Tumor cells and surface epithelium in ovaries with tumors were variably stained. Overall the expression of S1P1 in hen and human ovaries and in the ovarian tumors examined was remarkably similar.

Previous reports of S1P1 detected in Western blots indicated various molecular sizes [41], although the expected size is 47 kDa [2]. We observed a 47 kDa species by Western blotting in a membrane-enriched fraction, although it was often faint or undetectable. However, there were two predominant higher molecular weight species (72 and 108 kDa); these are not usually described although they are evident in some reports [42]. Notably, the same molecular sizes were observed in hen and human ovaries and ovarian tumors, hen spleen, and hen and rat brain. Because bands react with S1P1 antibody, the larger size bands may represent aggregates in dimmers or trimers [43]. Alternatively, S1P1 receptor may also be differentially glycosylated [44]. Nonetheless, similar protein bands were detected in the human and hen ovary, demonstrating a similar expression pattern.

The immunohistochemical pattern of S1P1 staining was common to both hen and human ovaries. Normal hen ovary expressed S1P1 in surface epithelial cells, theca cells of the follicle, endothelial cells of blood vessels in the stroma and medullary region, as well as in immune cells such as infiltrating immune cells of atretic follicles. The expression of S1P1 was not confined to immune cells. Because the human ovaries used in this study were from older women, they did not have any follicles for comparison. However, S1P1 was similarly expressed in endothelial cells and immune cells. It is unclear if S1P1 is expressed on the surface epithelium of human ovarian tumors, because many of the human ovarian tumor specimens obtained after diagnostic pathology did not have intact surface epithelium. However, in hen and human ovarian tumors S1P1 was expressed in endothelial cells and immune cells. In addition, tumors cells expressed S1P1 and the expression was dispersed throughout the cytoplasm. Furthermore, S1P1 expression varied among ovarian tumors. This may have been due to variations in expression among tumors or among tumor types or to sampling of individual tumors.

## Conclusion

In summary, S1P1 is expressed on immune cells in the hen. S1P1 is also expressed in ovarian tissues of the laying hen with a distribution in the ovary that is similar to human ovaries. The chicken embryo contains both sphingosine-1 phosphate (ligand for S1P1) and sphingosine kinase; the enzyme responsible for the conversion of sphingosine to sphingosine-phosphate which occurs in the blood [45]. Similarly, chicken embryonic amacrine cells were recently reported to express S1P1 [46], indicating that this receptor can be found in both embryonic and, as our study shows, the adult tissues of the chicken.

We also show, for the first time, that S1P1 is expressed in both hen and human ovarian tumors. S1P (the ligand for S1P1) has been implicated in the trafficking of immune cells [5]. Immune cells are reported to be involved in the progression of tumors of various organs [47]. While the role of infiltrating immune cells in ovarian cancer progression is not clearly defined [48] there clearly is a relationship of infiltrating T cells and survival [48-50]. The hen provides an alternative animal model to engineered rodent models for studies of ovarian cancer. Further studies addressing immune cell infiltration into tumors and the role S1P1 plays in regulating immune cell infiltration into ovarian tumors would be facilitated by use of the hen because all stages of spontaneous tumors in the hen can be readily observed.

## Competing interests

The authors declare that they have no competing interests.

## Authors' contributions

MJB carried out the molecular and immunohistochemical studies, participated in the sequence alignment, assisted in tissue collection, and drafted the manuscript. AB classified hen ovarian tumors, collected all tissue, prepared tissue for immunohistochemistry and maintained databases. YY reproduced the RT-PCR experiments. KP assisted in Western blotting experiments and maintained the tissue inventory. SLE designed PCR primers, prepared the sequence comparisons and assisted in the molecular biology experiments and their design. SS provided human ovarian tissue used in this study and participated in discussions of the experimental design. JSA assisted AB in detecting potential ovarian tumors with ultrasound. JMB maintained hens, collected tissue with AB and MJB and contributed expertise in avian physiology. JLL conceived the study, participated in its design and coordination, data analysis and preparation of the manuscript with MJB. All authors read and approved the final manuscript
